# Supplementary material for: Structural basis of Acinetobacter type IV pili targeting by an RNA virus
Source: Nat Commun. 2024 Mar 29;15:2746. doi: 10.1038/s41467-024-47119-5 (PMC10980823; doi:10.1038/s41467-024-47119-5)
Supplement: Supplementary file 1 — Supplementary Information [file 41467_2024_47119_MOESM1_ESM.pdf]

Supplementary Information

Structural basis of *Acinetobacter* type IV pili targeting by an RNA virus

Ran Meng[1,3,#], Zhongliang Xing[1,#], Jeng-Yih Chang[1,4,#], Zihao Yu[1], Jirapat Thongchol[1], Wen Xiao[1], Yuhang Wang[1], Karthik Chamakura[1,5], Zhiqi Zeng[1], Fengbin Wang[2], Ry Young[1], Lanying Zeng[1], Junjie Zhang[1,*]

[1]Center for Phage Technology, Department of Biochemistry and Biophysics, Texas A&M University, College Station, TX 77843, USA

[2]Department of Biochemistry and Molecular Genetics, Heersink School of Medicine, University of Alabama at Birmingham, Birmingham, AL 35294, USA

[3]Present address: Yale University, New Haven, CT 06520, USA

[4]Present address: UMass Chan Medical School, Worcester, MA 01655, USA

[5]Present address: Armata Pharmaceuticals, Inc., Marina del Rey, CA 90292, USA

[#]These authors contributed equally to this work

[*]Correspondence author. Email: junjiez@tamu.edu (J. Z.)

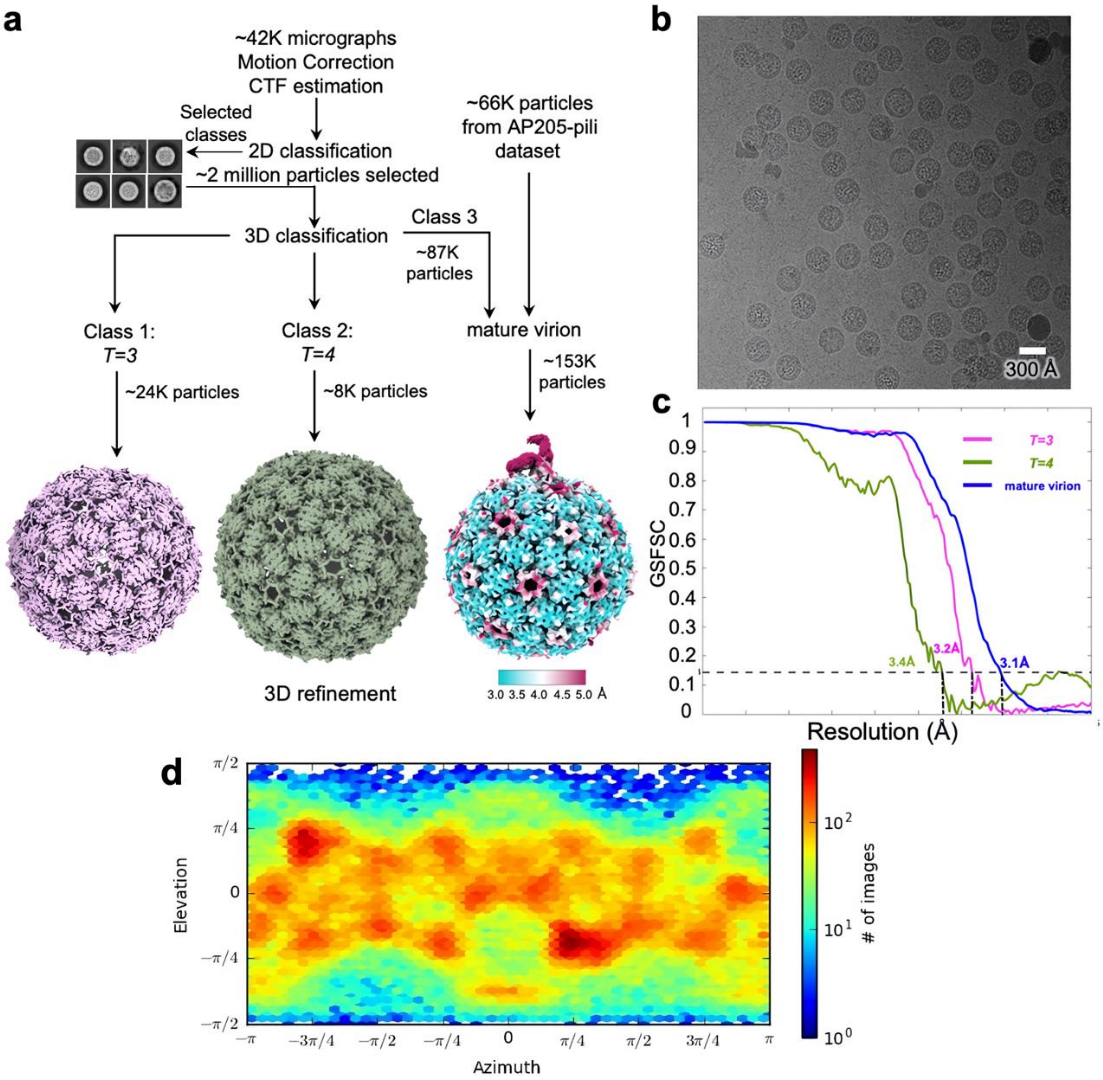

Supplementary Fig. 1. Cryo-EM data processing of AP205 virions and VLPs. a Cryo-EM data processing pipeline of the AP205 apo state with density maps colored lavender for the *T=3* capsid (bottom left), green for the *T=4* capsid (bottom middle), and colored by local resolutions for the mature virion (bottom right). **b** A representative micrograph of the AP205 particles. The scale bar denotes 300 Å. **c** Gold-Standard Fourier Shell Correlation (GSFSC) plot for cryo-EM reconstructions of the AP205 *T=3* VLP, *T=4* VLP, and the mature virion. **d** Angular distribution of the particles used to reconstruct the mature AP205 virion Cryo-EM density map.

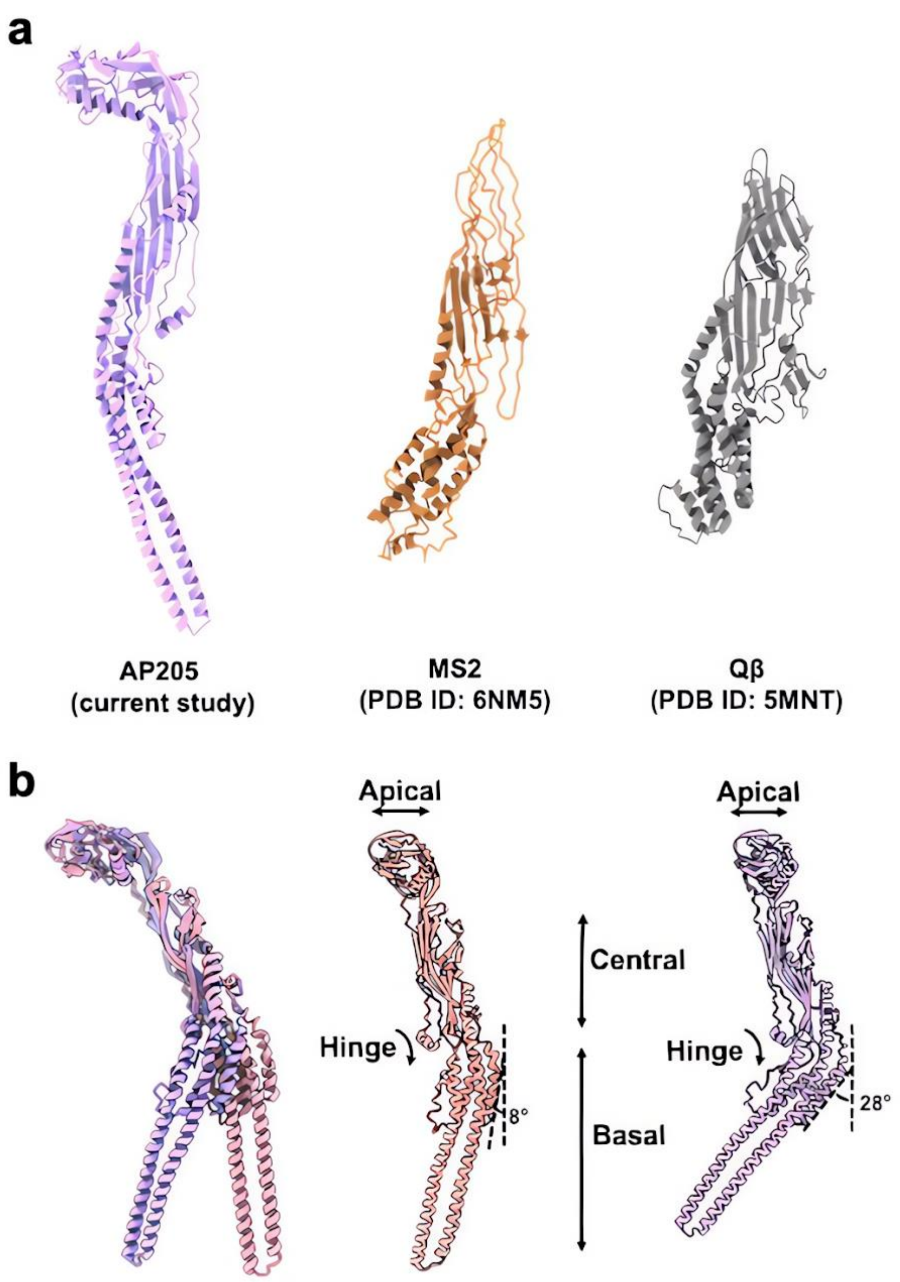

Supplementary Fig. 2. Comparison of Mat models. a Comparison of Mat structures from ssRNA phage AP205, MS2 and Qβ. **b** Comparison of the inner (purple) and outer (pink) Mat of AP205, with apical, central, and basal domains labeled. Notably, the angles between the central and basal domains differ between the two Mat of each AP205 virion.

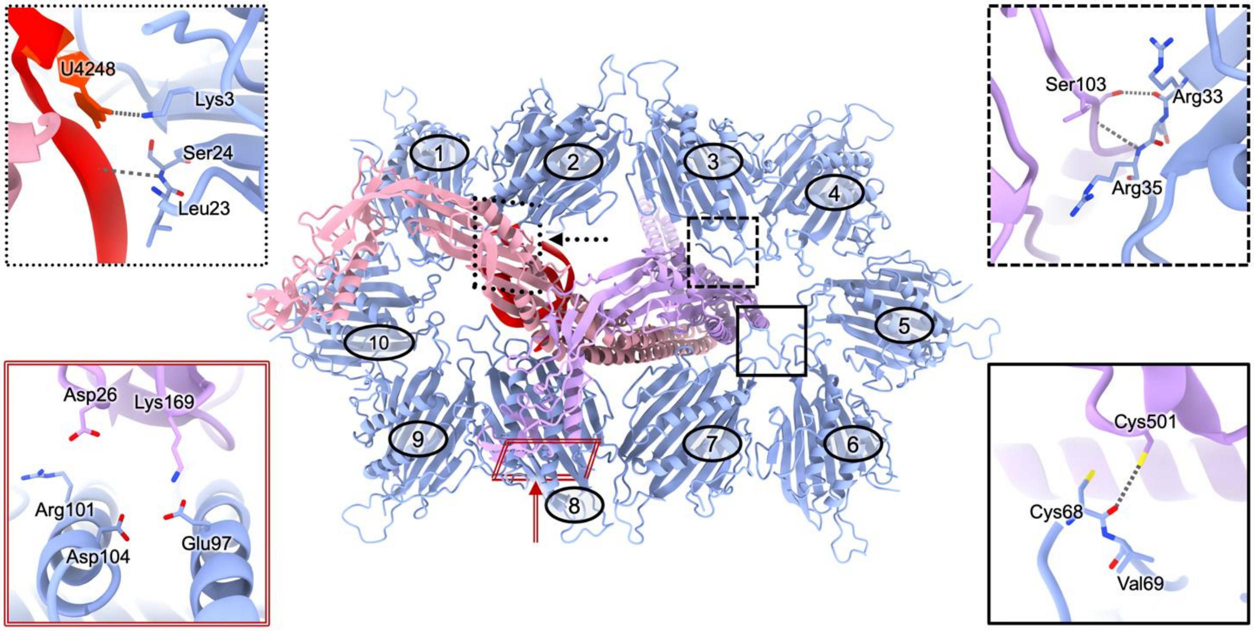

Supplementary Fig. 3. Interaction between the Mat-dimer and the Coat shell. The central panel illustrates the Mat-dimer surrounded by ten copies of Coat-dimers, numbered from 1 to 10. Red and black arrows indicate the viewing directions. Subpanels, at the four corners, depict the interactions between the Coat and Mat-dimer, which occurred in the corresponding boxes labeled on the model in the central panel. These interactions include: The 3' end of the gRNA being sandwiched between the outer Mat and Coat-dimer 2 (Top left subpanel); Coat-dimer 3 presents Arg33 and Arg35 to interact with inner Mat residue Ser103 through hydrogen bonds (top right subpanel); Local electrostatic interaction between Coat-dimer 8 residues Arg101, Asp104, and Glu97 and inner Mat residues Asp26 and Lys169 (bottom left subpanel); Hydrogen bonding between inner Mat residue Cys501 and the backbone oxygen of Cys68 on Coat-dimer 7 (bottom right subpanel).

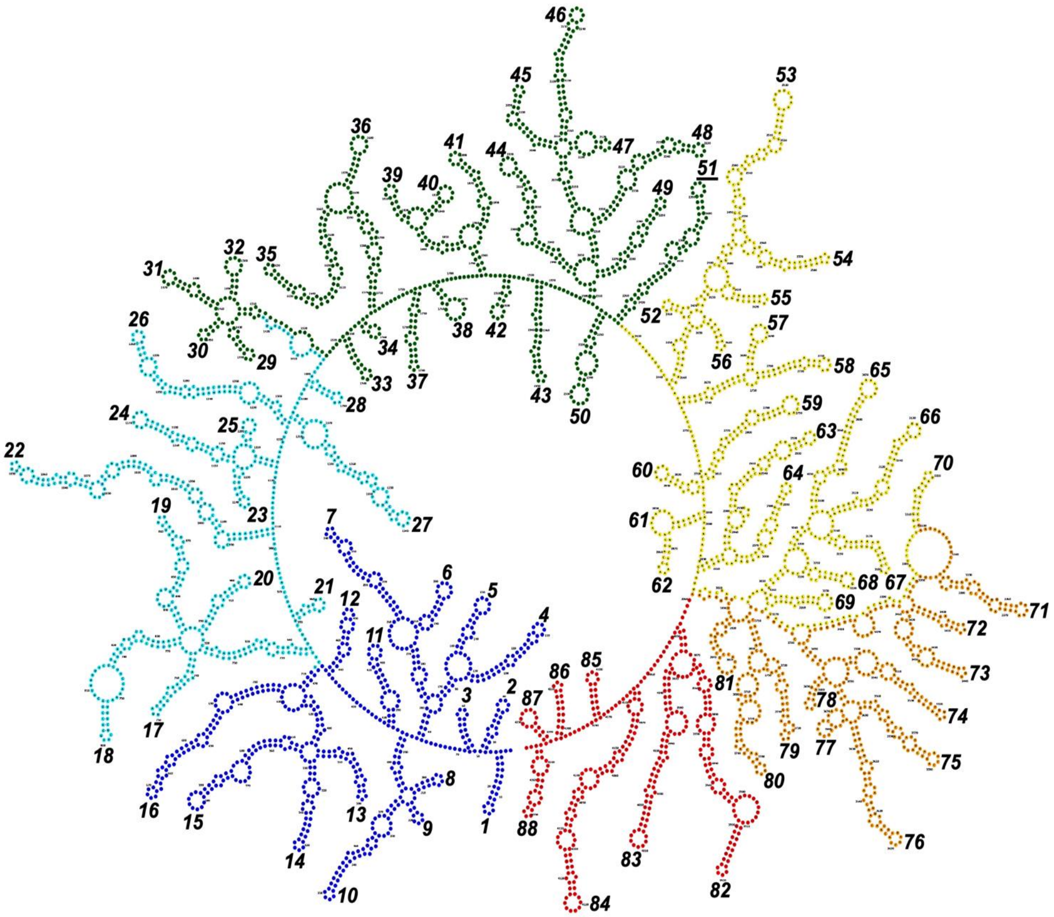

Supplementary Fig. 4. Secondary structure of the AP205 gRNA. The secondary structure of the entire 4269 nucleotides of the AP205 genome, visualized by Forna[1], is colored in rainbow. The numbers label the 88 stem-loops in the AP205 genome.

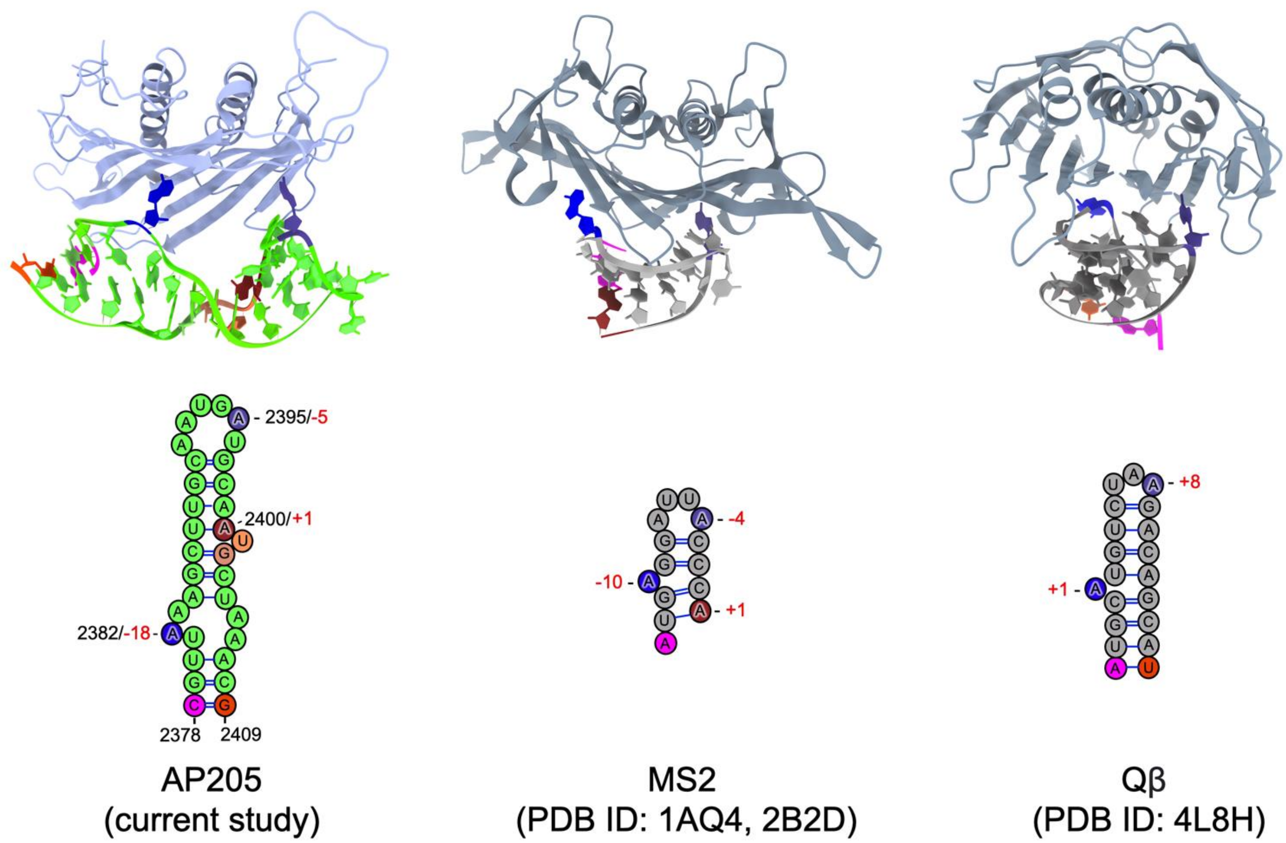

Supplementary Fig. 5. Differences among the operators in AP205, MS2, and Qβ. Top row: interactions with the Coat-dimer. Bottom row: secondary structures of the operators. The numbers in red annotate the relative positions of corresponding residues to the start codon (AUG) of the *rep* gene.

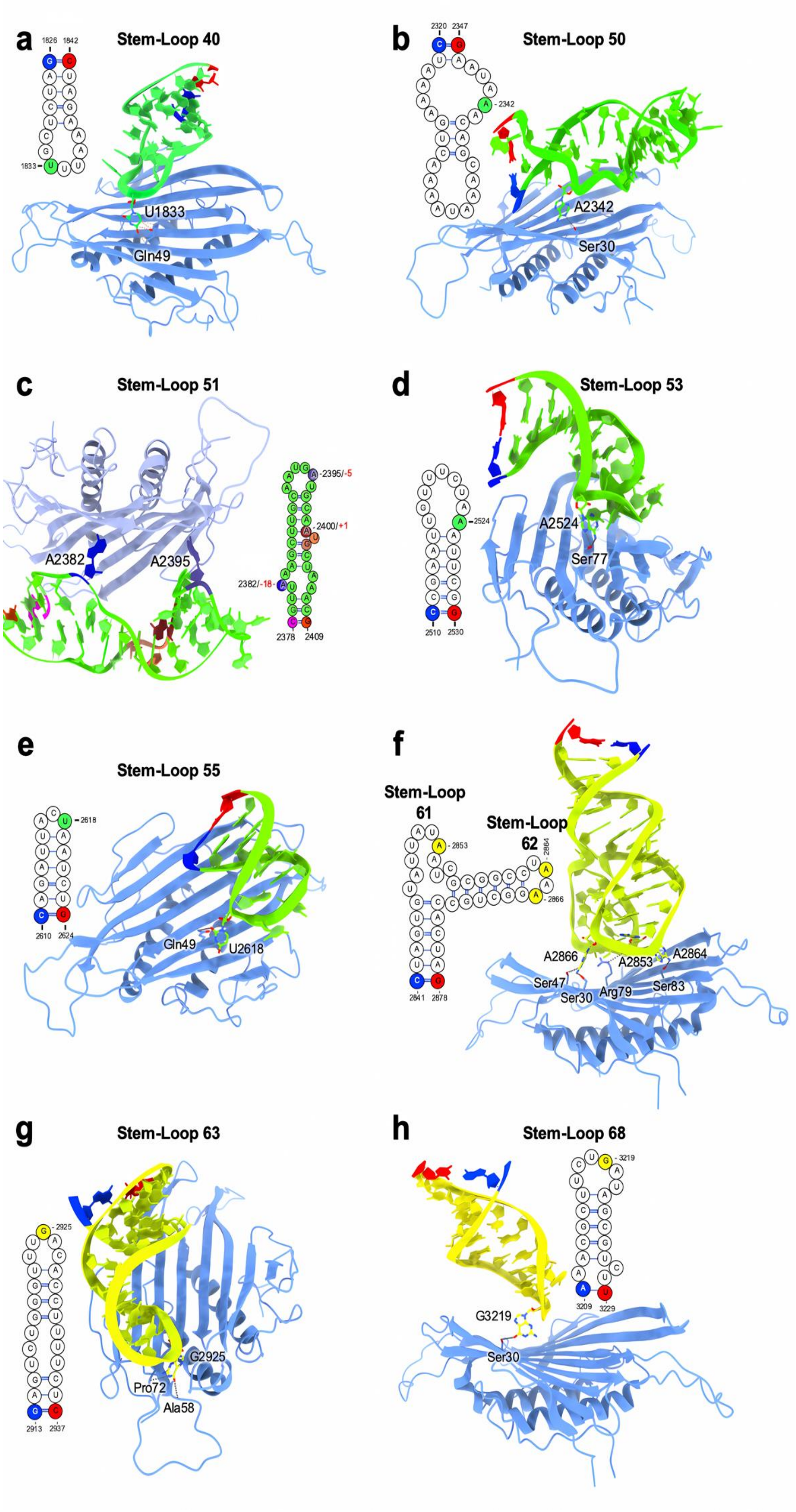
a
Stem-Loop 40
U1833
Gln49
b
Stem-Loop 50
A2342
Ser30
c
Stem-Loop 51
A2382
A2395
d
Stem-Loop 53
A2524
Ser77
e
Stem-Loop 55
Gln49
U2618
f
Stem-Loop 61
Stem-Loop 62
A2866
A2853
A2864
Ser47
Ser30
Arg79
Ser83
g
Stem-Loop 63
G2925
Pro72
Ala58
h
Stem-Loop 68
G3219
Ser30

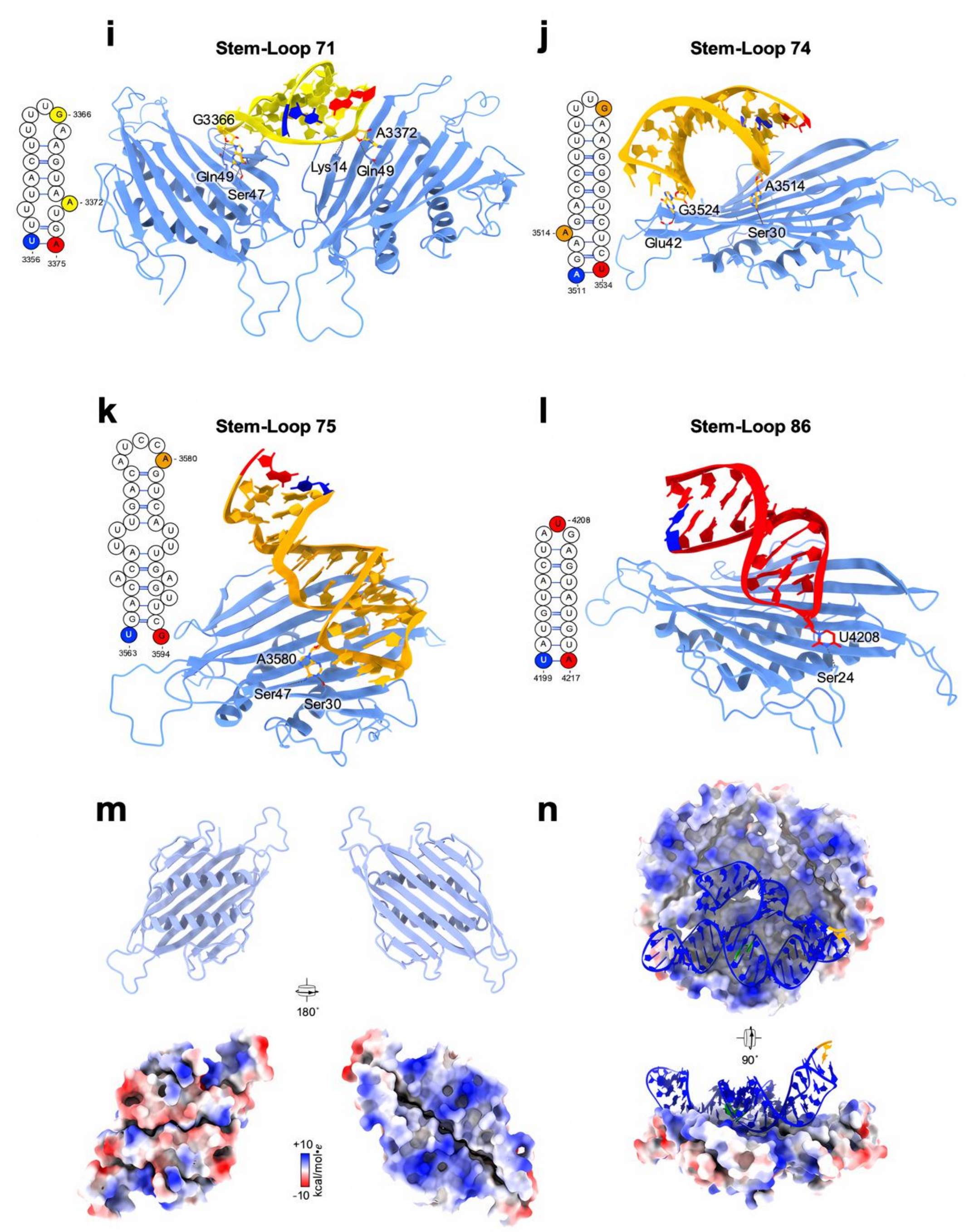

Supplementary Fig. 6. Examples of gRNA-Coat interactions in the AP205 virion. a-l The thirteen RNA stem-loops which specifically interact with the Coat-dimers. The RNA secondary structures of these stem-loops are shown on the side. **m** The model and electrostatic potential surface of a Coat-dimer viewing from outside (left column) or inside (right column) of the capsid. **n** Two orthogonal views of the model for the first 75 nucleotides of the gRNA at the 5' end interacting with three Coat-dimers. The Coat-dimers are shown as electrostatic potential surface.

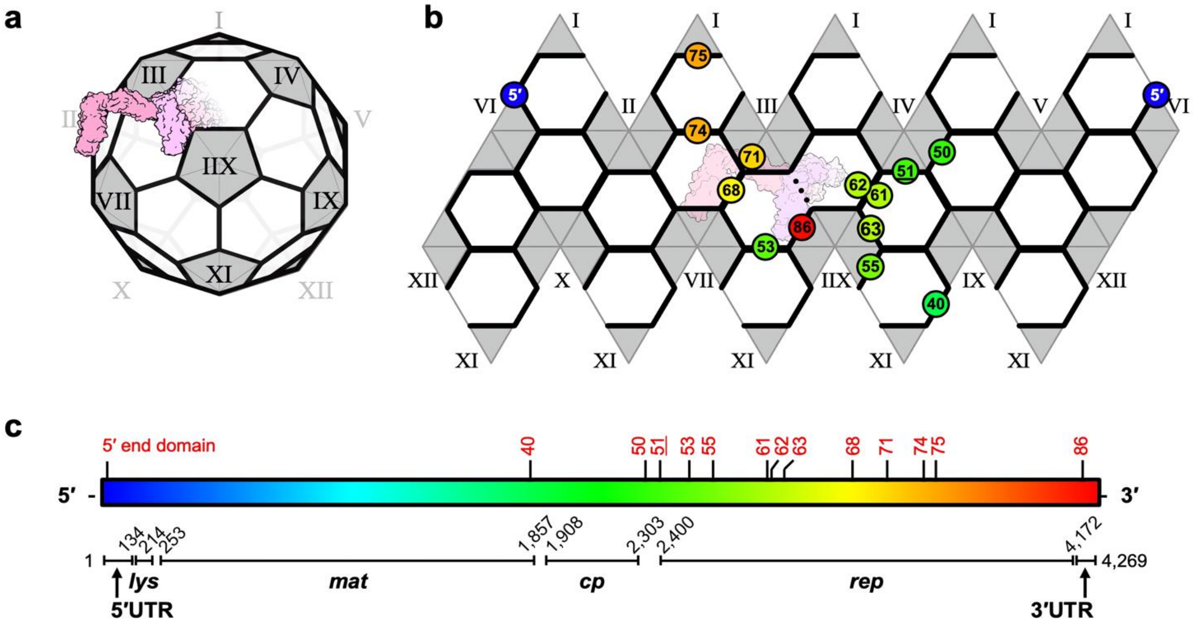

Supplementary Fig. 7. Locations of the RNA stem-loops that specifically interact with the Coat shell. a Cage model for the AP205 virion showing pentameric (gray) and hexameric (white) faces on the capsid. Each pentameric facet was labeled with Roman numerals from I-XII. Each thick solid black line represents a Coat-dimer. The Mat-dimer is located between pentameric faces III and IIX. **b** an unwrapped AP205 cage lattice into 2D with each thick solid black line represents a Coat-dimers on the capsid, while the thick dotted line represents the location of the Coat-dimer that was replaced by the Mat-dimer. The thirteen RNA stem-loops that specifically interact with Coat-dimers are circled and labeled on the 2D lattice. The Mat-dimer is shown as transparent to aid visualization of those stem-loops interacting with the Coat shell. Stem-loops 61 and 62 interact with the same Coat-dimer. Stem-loop 71 interacts with two Coat-dimers. The RNA domain formed by the first 75 nts is also labeled (5’, blue circle) and interacts with three Coat-dimers. c The diagram of the AP205 genome colored rainbow from the 5′ end (blue) to the 3′ end (red). The core genes: *lys*, *mat*, *coat(cp)*, and *rep* are denoted under the diagram. The red numbers denote the thirteen RNA stem-loops and the 5’ end domain plotted in panel **b**. The underlined stem-loop 51 is the actual RNA translational operator for the *rep* gene. See Supplementary Fig. 6 for the sequence and model for each of these RNA stem-loops and domains.

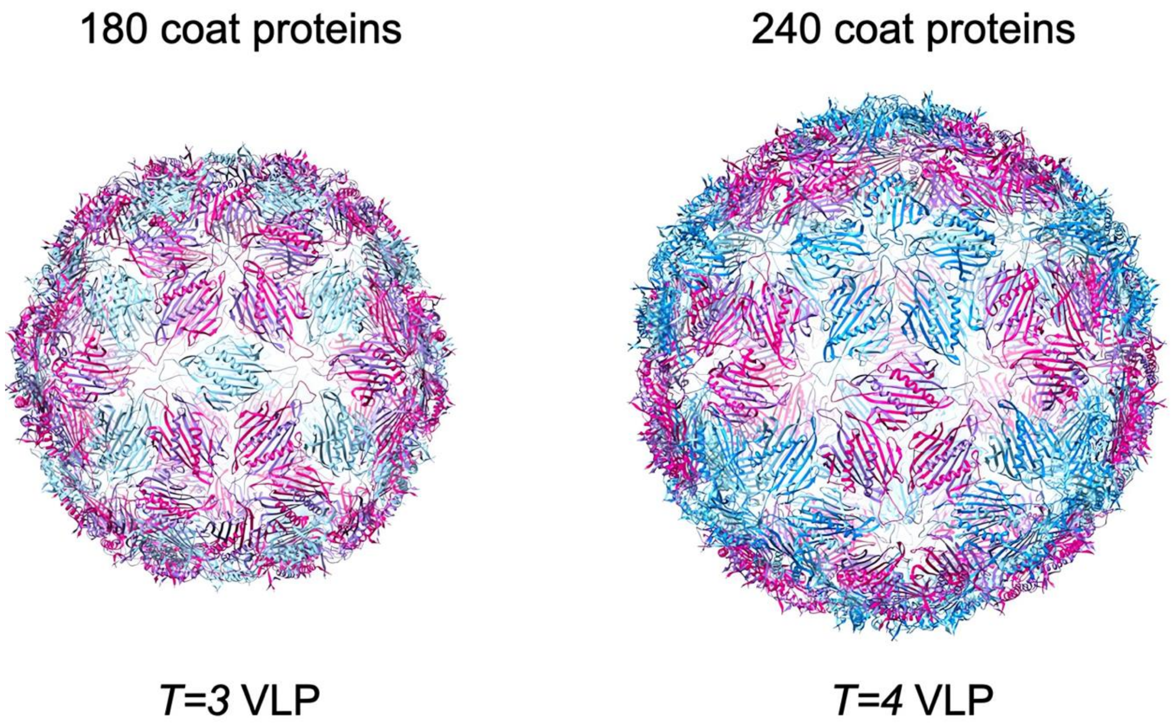

Supplementary Fig. 8. Structures of AP205 *T=3* and *T=4* VLPs. The *T=3* (left) and *T=4* (right) VLP capsids are assembled by 180 and 240 copies of Coat, respectively.

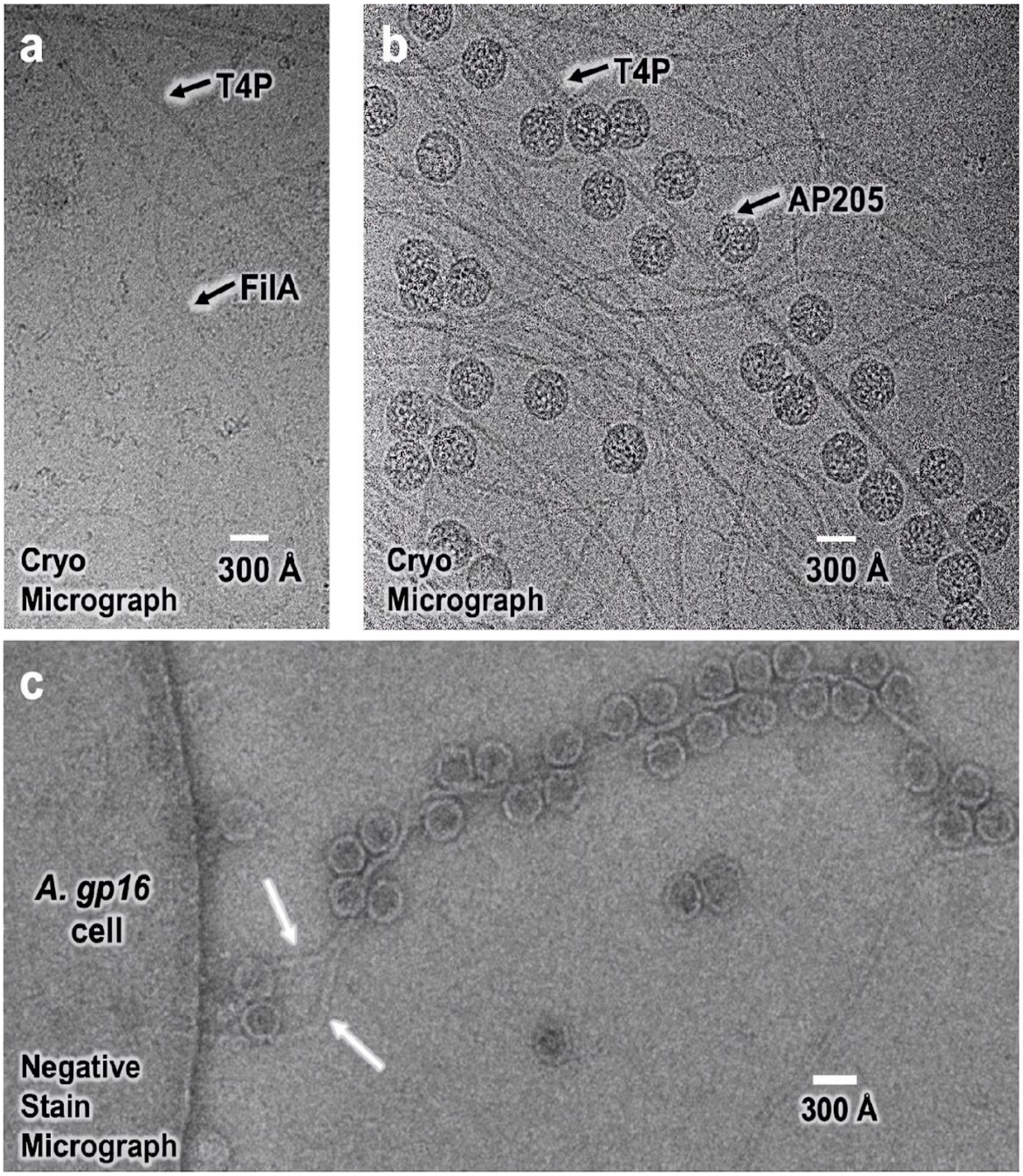

Supplementary Fig. 9. AP205 only binds to T4P instead of FilA pili. a Cryo-EM shows T4P and FilA pili (No phage added). **b** Cryo-EM shows each AP205 can bind to one or two straight T4P. FilA pili are curved, without any phage bound. **c** Negative-stain EM shows phages adsorb to two T4P *in situ*. Here T4P are curved due to negative staining. White arrows indicate two T4P emanating from two pilus-assembly machines at the cell envelope. The scale bars denote 300 Å in all micrographs.

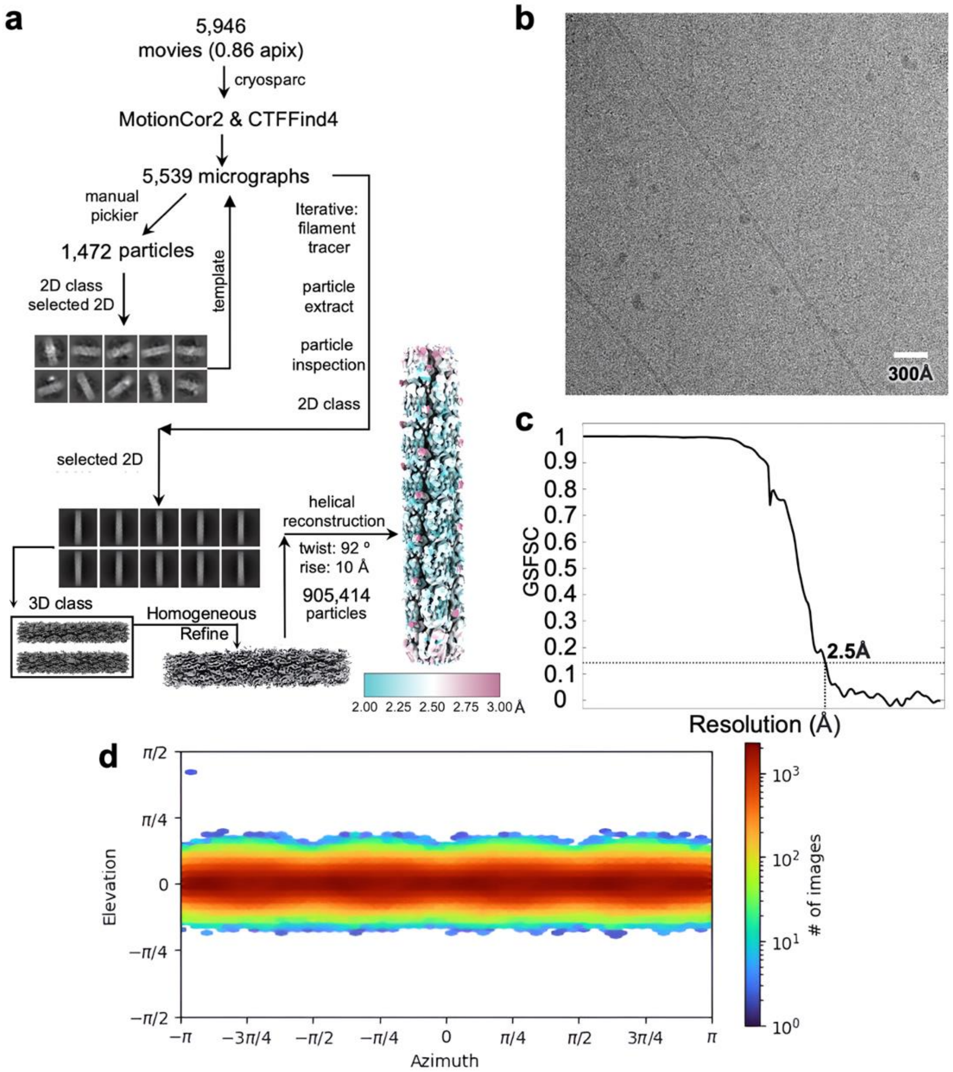

Supplementary Fig. 10. Cryo-EM data processing of *A. gp16* T4P. a The cryo-EM data processing pipeline for the T4P is illustrated, with the final density map (lower right) colored based on local resolutions. **b** A region of an exemplary micrograph from the T4P dataset. T4P is straight in vitreous ice. The scale bar denotes 300 Å. **c** The resolution for the T4P reconstruction is estimated by the Gold-Standard Fourier Shell Correlation (GSFSC) plot. **d** Angular distribution of the particles used to reconstruct the *A. gp16* T4P Cryo-EM density map.

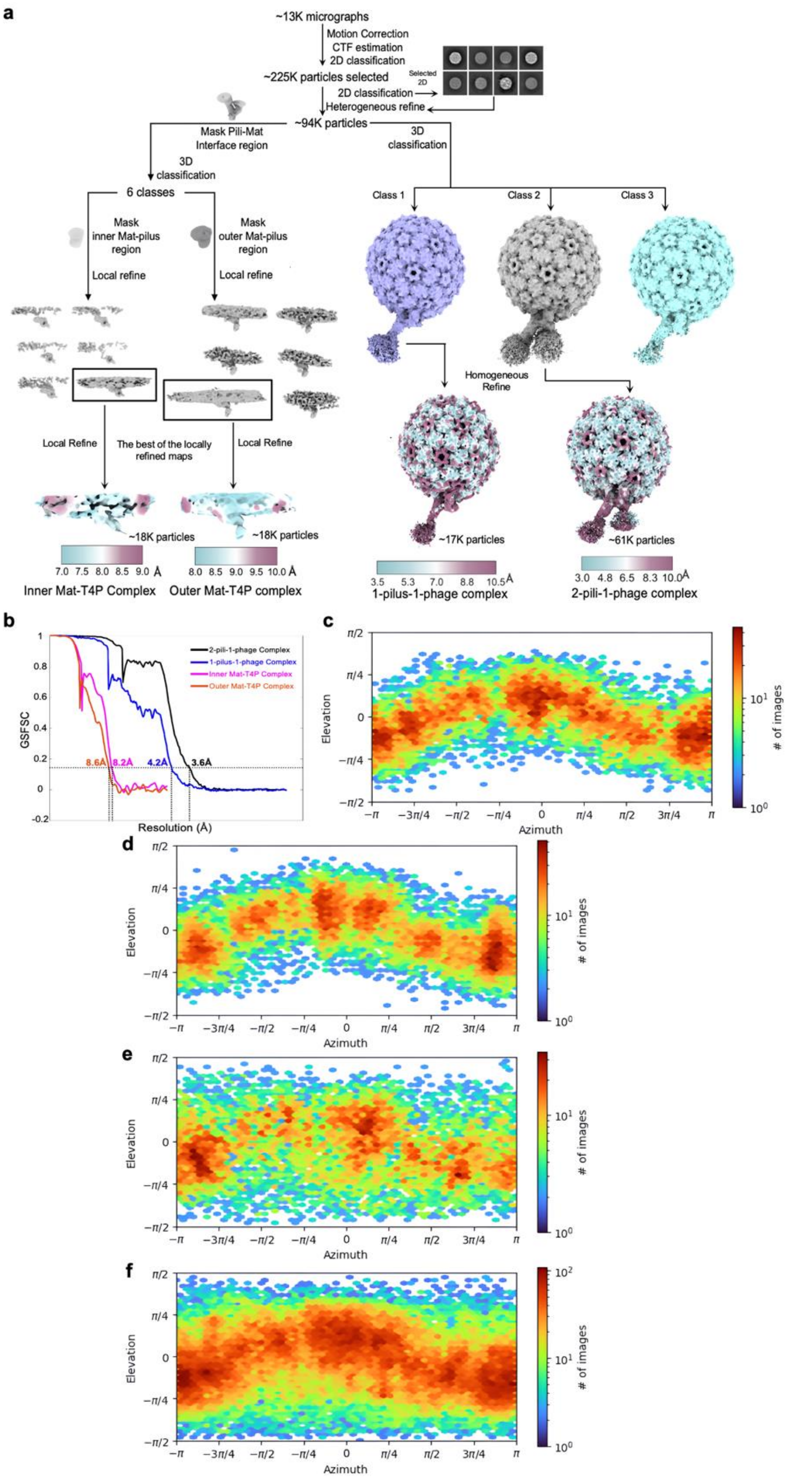

Supplementary Fig. 11. Cryo-EM data processing of the AP205-T4P complex. a The cryo-EM data processing pipeline for the AP205-T4P complex is depicted, with the final density maps colored based on local resolutions (bottom row). **b** The Gold-Standard Fourier Shell Correlation (GSFSC) plot estimating resolutions for the reconstructions. **c-f** Angular distributions of the particles used to reconstruct the Cryo-EM density maps for Inner Mat-T4P, Outer Mat-T4P, 1-pilus-1-phage, and 2-pili-1-phage, respectively.

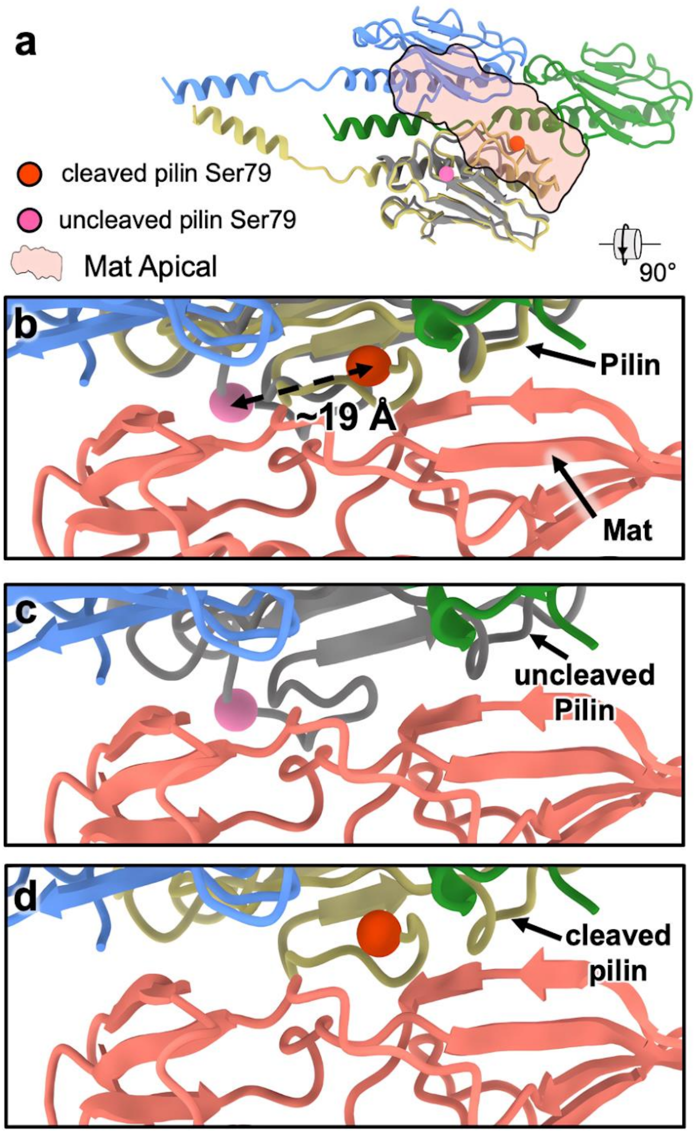

Supplementary Fig. 12. AP205 Mat is adapted to the internally cleaved pilin. a The cleaved pilins are colored blue, green, and yellow for Pilin 1, 2, and 3, respectively, as in Fig. 4. The internal cleavage site at Ser79 of Pilin 3 is at the Mat-binding interface. The AlphaFold-modeled uncleaved Pilin 3 (gray) is overlaid onto the cleaved Pilin 3 (yellow). The "footprint" of Mat is colored salmon. The red sphere represents Ser79 of the cleaved pilin from the native T4P, while the pink sphere represents Ser79 of the uncleaved pilin modeled by AlphaFold. **b** Viewing the binding interface between Mat and Pilin 3. In the absence of the cleavage at Ser79, the interface would adopt a different conformation with Ser79 being ~19Å away from its current location, which can be incompatible for Mat binding. c and d The interface between Mat and pilins if they were not post-translationally cleaved (Panel **c**), or if they are cleaved at Ser79 (Panel **d**).

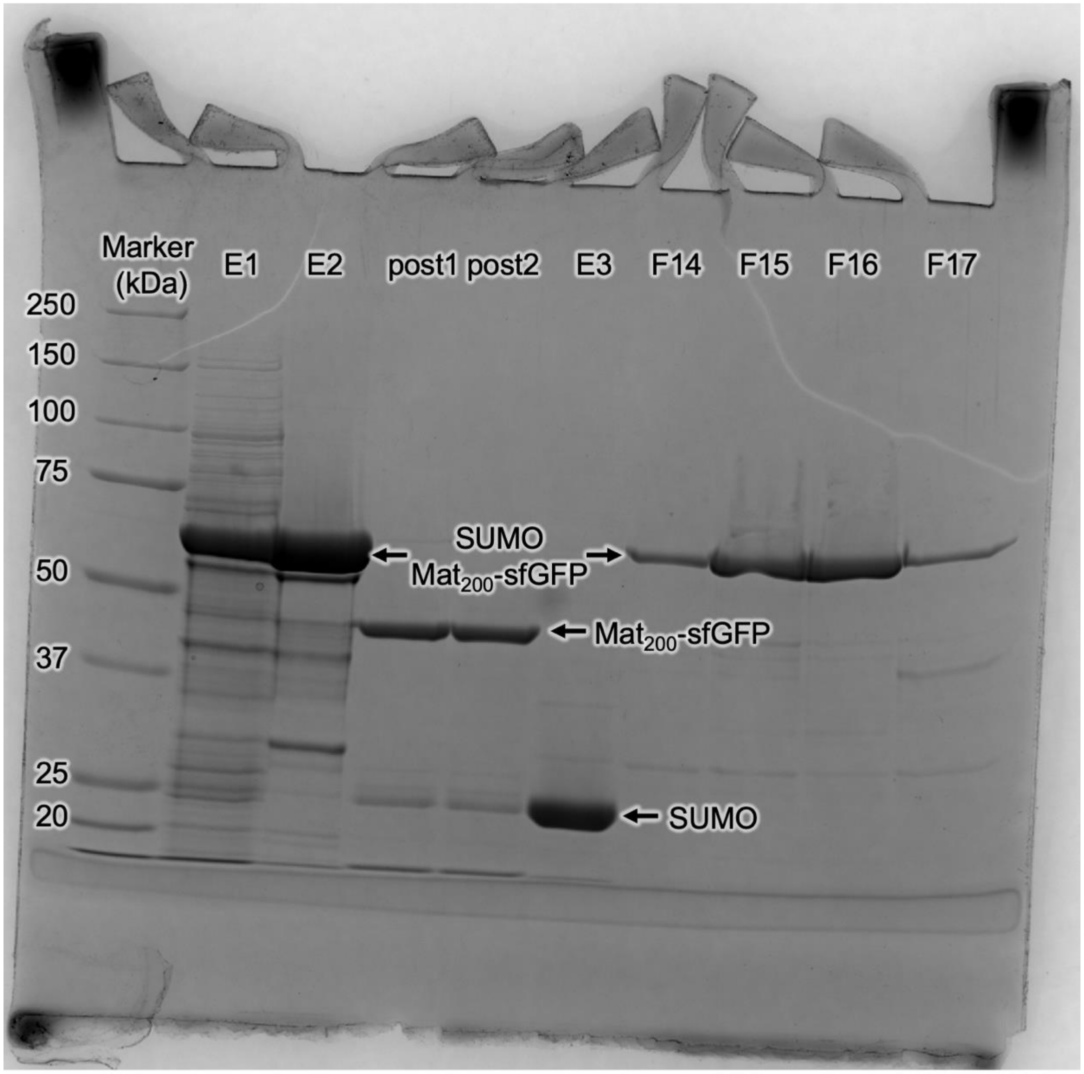

Supplementary Fig. 13. SDS PAGE gel for SUMO-Mat200-sfGFP Purification. Lane E1 & E2: eluted SUMO-Mat200-sfGFP using 100 mM and 300 mM Imidazole, respectively. Lane post1 and post 2: after the sumo protease cleavage reactions, when sumo tags were cleaved, leaving Mat200-sfGFP. Lane E3: eluted SUMO protein using 500 mM Imidazole. Lane F14-F17: eluted SUMO-Mat200-sfGFP protein fractions from a Superdex 200 size-exclusion column.

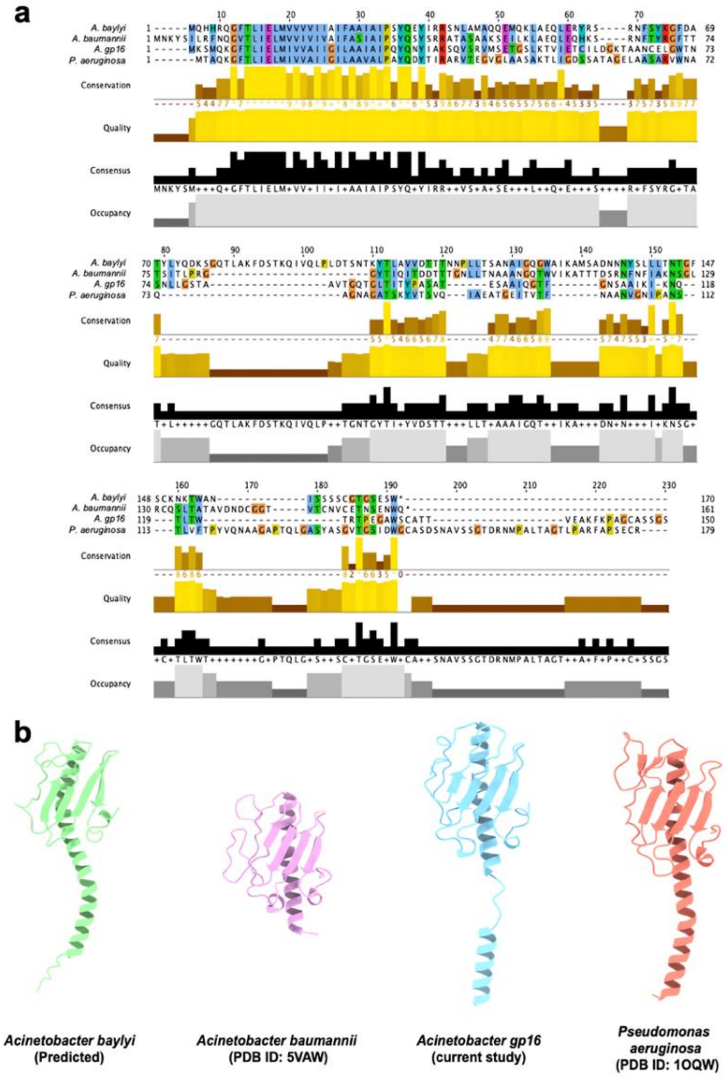

Supplementary Fig. 14. Comparison of type IV pilins from different bacteria. a Sequence and **b** Structure comparison of type IV pilins from *Acinetobacter baylyi*, *Acinetobacter baumannii*, *Acinetobacter gp16*, and *Pseudomonas aeruginosa*. The four pilins have relatively conserved N-terminal sequences though the C-termini are not conserved. The overall pilin structures are similar and consist of an α-helical domain and a β-sheet domain, forming a 'lollipop' shape. The color in panel A follows the JalView[2] default color scheme with each residue in the alignment assigned a color if the amino acid profile of the alignment at that position meets minimum criteria specific for the residue type (Hydrophobic, Blue; Positive charge, Red; Negative charge, Magenta; Polar, Green; Cysteine, Pink; Glycine, Orange; Prolines, Yellow; Aromatic, Cyan; Unconserved, White. The yellow and black bars represent conservation, alignment quality and consensus scores. The gray occupancy bars represent numbers of aligned positions).

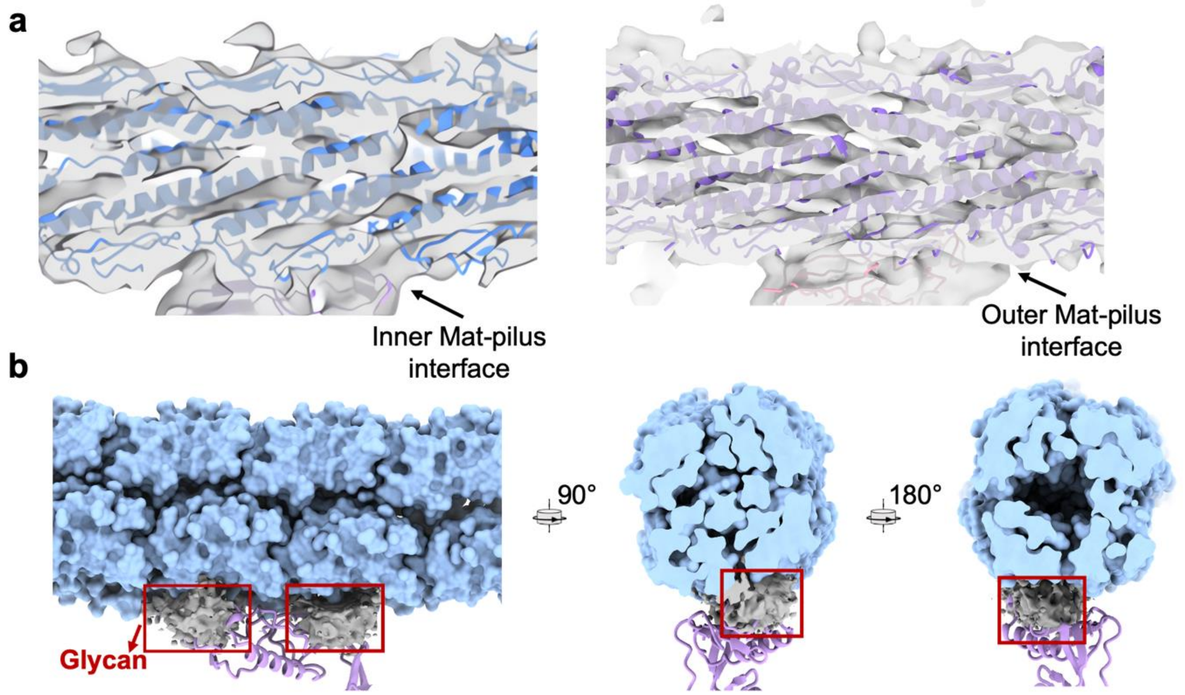

Supplementary Fig. 15. Models of the Mat-T4P complex fitted into the locally refined cryo-EM density for T4P bound to the inner and outer Mat, respectively. a The atomic models of inner and outer Mat-pilus complexes are shown fitted into their respective locally refined cryo-EM density maps. Black arrows indicate the Mat-pilus interfaces. **b** The model of Inner Mat-pilus complex is fitted into the reconstructed *A. gp16* T4P Cryo-EM density map. Glycan densities (gray) from the Cryo-EM map are highlighted in red boxes. It is observed that the presence of glycans on the T4P will interfere with the binding of the Mat (purple model).

Supplementary Table 1. Cryo-EM data collection, refinement, and validation statistics.

	T4P (EMDB-41442) (PDB 8TOB)	AP205 (EMDB-41443) (PDB 8TOC)	Inner Mat-T4P (EMDB-41634) (PDB 8TV9)	Outer Mat-T4P (EMDB-41635) (PDB 8TVA)	T=3 (EMDB-41666) (PDB 8TWC)	T=4 (EMDB-41657) (PDB 8TW2)	2-T4P-1-AP205 (EMDB-41646)	1-T4P-1-AP205 (EMDB-41447)
**Data collection and processing**								
Magnification	105,000	130,000	130,000	130,000	130,000	130,000	130,000	130, 000
Voltage (kV)	300	300	300	300	300	300	300	300
Electron exposure (e–/Å^2)	50	50	50	50	50	50	50	50
Defocus range (μm)	-2.0 – 2.0	-3.5 – -1.0	-3.5 – -1.0	-3.5 – -1.0	-3.5 – -1.0	-3.5 – -1.0	-3.5 – -1.0	-3.5 – - 1.0
Pixel size (Å)	0.86	1.06	1.06	1.06	1.06	1.06	1.06	1.06
Symmetry imposed	Helical	C1	C1	C1	C1	C1	C1	C1
Initial particle images (no.)	5,946	41,468	12,703	12,703	41,468	41,468	12,703	12,7 03
Final particle images (no.)	5,946	41,468	12,703	12,703	41,468	41,468	12,703	12,7 03
Map resolution (Å)	2.5	3.1	8.2	8.6	3.2	3.4	3.6	4.2
FSC threshold	0.143	0.143	0.143	0.143	0.143	0.143	0.143	0.14 3
Map resolution range (Å)	2.0 – 3.0	3.0 – 5.0	7.0 – 9.0	8.0 – 10.0	N/A	N/A	3.0 – 10.0	3.5 – 10.5
**Refinement**								
Initial model used (PDB code)	AlphaFold	Coat (5LQP) Mat (I-TASSER) RNA (ab initio)	This Study	This Study	5LQP	5LQP		
Model composition								
Non-hydrogen atoms	22,242	271,353	22,502	24,524	174,240	232,320		
Protein residues	3,058	24,030	3,036	3,314	23,220	30,960		
R.m.s. deviations								
Bond lengths (Å)	0.002	0.004	0.005	0.003	0.003	0.005		
Bond angles (°)	0.449	0.609	0.843	0.720	0.645	0.678		
Validation								
MolProbity score	1.41	2.11	2.50	2.27	1.46	1.89		
Clashscore	4.48	15.11	13.03	10.48	5.28	13.36		
Poor rotamers (%)	0.00	0.00	0.00	0.00	0.05	0.00		
Ramachandran plot								
Favored (%)	96.87	93.48	95.71	95.88	96.96	96.24		
Allowed (%)	3.06	5.76	4.09	4.02	2.78	3.76		
Disallowed (%)	0.07	0.76	0.20	0.09	0.75	0.00		

Supplementary Table 2. Information on the materials used in the research.

Materials	Catalogue No.	Vendor/Source	Sequence
His SUMO Mat_{200} sfGFP DNA	N/A	GenScript Biotech®	Please see Source Data File 1.
pET 28a+ vector	N/A	GenScript Biotech®	Please see Source Data File 1.
A. gp16 cell	17988	ATCC®	N/A
E. coli BL21 DE3 cell	C2527H	New England Biolabs®	N/A

Supplementary References

1. Kerpedjiev, P., Hammer, S. & Hofacker, I. L. Forna (force-directed RNA): Simple and effective online RNA secondary structure diagrams. *Bioinformatics* 31, 3377–3379 (2015).
2. Waterhouse, A. M., Procter, J. B., Martin, D. M. A., Clamp, M. & Barton, G. J. Jalview Version 2—a multiple sequence alignment editor and analysis workbench. *Bioinformatics* 25, 1189–1191 (2009).
