# Supplementary material for: Structural basis of Acinetobacter type IV pili targeting by an RNA virus
Source: Nat Commun. 2024 Mar 29;15:2746. doi: 10.1038/s41467-024-47119-5 (PMC10980823; doi:10.1038/s41467-024-47119-5)
Supplement: Supplementary file 3 — Description of Additional Supplementary Files [file 41467_2024_47119_MOESM3_ESM.pdf]

Description of Additional Supplementary Files

File Name: Supplementary Data 1
Description: AlphaFold predicted A. gp16 T4P pilin model.

File Name: Supplementary Movie 1
Description: The complete structure of AP205 virion features a Mat-dimer and conserved π-stacking between protein side-chains and RNA bases.

File Name: Supplementary Movie 2
Description: Morphing between the conformations of outer and inner Mat of AP205.

File Name: Supplementary Movie 3
Description: The electrostatic potential surface of AP205 outer Mat.

File Name: Supplementary Movie 4
Description: The electrostatic potential surface of AP205 inner Mat.

File Name: Supplementary Movie 5
Description: The electrostatic potential surface of AP205 Mat-dimer.
