# Supplementary material for: Structural basis of Acinetobacter type IV pili targeting by an RNA virus
Source: Nat Commun. 2024 Mar 29;15:2746. doi: 10.1038/s41467-024-47119-5 (PMC10980823; doi:10.1038/s41467-024-47119-5)
Supplement: Supplementary file 10 — Reporting Summary [file 41467_2024_47119_MOESM10_ESM.pdf]

nature portfolio

Corresponding author(s): Junjie Zhang

Last updated by author(s): Jan 26, 2024

# Reporting Summary

Nature Portfolio wishes to improve the reproducibility of the work that we publish. This form provides structure for consistency and transparency in reporting. For further information on Nature Portfolio policies, see our Editorial Policies and the Editorial Policy Checklist.

## Statistics

For all statistical analyses, confirm that the following items are present in the figure legend, table legend, main text, or Methods section.

| n/a | Confirmed | |
|---|---|---|
| ☐ | ☒ | The exact sample size (*n*) for each experimental group/condition, given as a discrete number and unit of measurement |
| ☐ | ☒ | A statement on whether measurements were taken from distinct samples or whether the same sample was measured repeatedly |
| ☒ | ☐ | The statistical test(s) used AND whether they are one- or two-sided<br>*Only common tests should be described solely by name; describe more complex techniques in the Methods section.* |
| ☒ | ☐ | A description of all covariates tested |
| ☒ | ☐ | A description of any assumptions or corrections, such as tests of normality and adjustment for multiple comparisons |
| ☒ | ☐ | A full description of the statistical parameters including central tendency (e.g. means) or other basic estimates (e.g. regression coefficient) AND variation (e.g. standard deviation) or associated estimates of uncertainty (e.g. confidence intervals) |
| ☒ | ☐ | For null hypothesis testing, the test statistic (e.g. *F*, *t*, *r*) with confidence intervals, effect sizes, degrees of freedom and *P* value noted<br>*Give P values as exact values whenever suitable.* |
| ☒ | ☐ | For Bayesian analysis, information on the choice of priors and Markov chain Monte Carlo settings |
| ☒ | ☐ | For hierarchical and complex designs, identification of the appropriate level for tests and full reporting of outcomes |
| ☒ | ☐ | Estimates of effect sizes (e.g. Cohen's *d*, Pearson's *r*), indicating how they were calculated |

*Our web collection on statistics for biologists contains articles on many of the points above.*

## Software and code

Policy information about availability of computer code

| | |
|---|---|
| Data collection | ThermoFisher EPU 1.2&2.1 for cryo-em data collection. |
| Data analysis | EMAN2 and Gautomatch are used to do particle picking. MotionCor2 is used to do movie corrections.Gctf is used to do some CTF estimation correction. Relion 3.0 and Cryosparc 3.0&4.0 are used to generate cryo-em density maps. I-TASSER and AlphaFold 2.2 are used to generate some initial models. Coot 0.8&0.9 and Chimerax 1.5 ISOLDE along Chimera 1.16 are used manually to modify protein models. Rosetta software package is used for RNA modeling. MDFF, RosettaCM, and Phenix 1.20.1 is used to refine models. |

For manuscripts utilizing custom algorithms or software that are central to the research but not yet described in published literature, software must be made available to editors and reviewers. We strongly encourage code deposition in a community repository (e.g. GitHub). See the Nature Portfolio guidelines for submitting code & software for further information.

## Data

Policy information about availability of data

All manuscripts must include a data availability statement. This statement should provide the following information, where applicable:

- Accession codes, unique identifiers, or web links for publicly available datasets
- A description of any restrictions on data availability
- For clinical datasets or third party data, please ensure that the statement adheres to our policy

The coordinates of A. gp16 T4P, AP205, inner Mat-T4P complex, outer Mat-T4P complex, AP205 T=4 VLP, and AP205 T=3 VLP are deposited in the Protein Data Bank (PDB) with the accession codes 8TOB [https://doi.org/10.2210/pdb8TOB/pdb], 8TOC [https://doi.org/10.2210/pdb8toc/pdb], 8TV9 [https://doi.org/10.2210/

pdb8tv9/pdb], 8TVA [https://doi.org/10.2210/pdb8tva/pdb], 8TW2 [https://doi.org/10.2210/pdb8tw2/pdb], and 8TWC [https://doi.org/10.2210/pdb8twc/pdb], respectively, as well as the referenced AP205 T=3 VLP has the PDB code 5LQP [https://doi.org/10.2210/pdb5LQP/pdb]. Cryo-EM maps of A. gp16 T4P alone, AP205 alone, inner Mat-T4P complex, outer Mat-T4P complex, AP205 T=4 VLP, AP205 T=3 VLP, AP205 bound to two T4P, and AP205 bound to one T4P are deposited in the Electron Microscopy Data Bank (EMDB) with the accession codes for EMD-41442 [https://www.ebi.ac.uk/emdb/EMD-41442], EMD-41443 [https://www.ebi.ac.uk/emdb/EMD-41443], EMD-41634 [https://www.ebi.ac.uk/emdb/EMD-41634], EMD-41635 [https://www.ebi.ac.uk/emdb/EMD-41635], EMD-41657 [https://www.ebi.ac.uk/emdb/EMD-41657], EMD-41666 [https://www.ebi.ac.uk/emdb/EMD-41666], EMD-41646 [https://www.ebi.ac.uk/emdb/EMD-41646], and EMD-41447 [https://www.ebi.ac.uk/emdb/EMD-41447], respectively.

## Research involving human participants, their data, or biological material

Policy information about studies with human participants or human data. See also policy information about sex, gender (identity/presentation), and sexual orientation and race, ethnicity and racism.

| | |
|---|---|
| Reporting on sex and gender | NA. |
| Reporting on race, ethnicity, or other socially relevant groupings | NA. |
| Population characteristics | NA. |
| Recruitment | NA. |
| Ethics oversight | NA. |

Note that full information on the approval of the study protocol must also be provided in the manuscript.

# Field-specific reporting

Please select the one below that is the best fit for your research. If you are not sure, read the appropriate sections before making your selection.

[x] Life sciences   [ ] Behavioural & social sciences   [ ] Ecological, evolutionary & environmental sciences

For a reference copy of the document with all sections, see nature.com/documents/nr-reporting-summary-flat.pdf

# Life sciences study design

All studies must disclose on these points even when the disclosure is negative.

| | |
|---|---|
| Sample size | 905,414 segments were used to generate the A. gp16 T4P resolution map; 1-phage-1-pilus map was generated using around 17K particles; 1-phage-2-pili map was generated using around 61K particles; inner-mat-pilus map was generated using around 18K particles; Outer-mat-pilus map was generated using around 18K particles; Around 153K particles were used to generate AP205 density maps; AP205 T=3 VLP density map was generated using around 24K particles; AP205 T=4 VLP density map was generated using around 8K particles. Around 42K movies were collected for AP205, 5,946 movies for A. gp16 T4P, and around 13K movies for AP205-T4P complex. Such sample sizes are chosen based on the fact that when more particles are added the sample, there is no improvement resolution-wise. |
| Data exclusions | All data sets were used to generate cryo-em density maps for the data presented in our manuscript. |
| Replication | With the large data set of the collected movies, we performed multiple data collections as to get the high resolution maps. In total, we got around 42K movies for the AP205 cryo-em dataset and around 13K movies for the AP205-T4P complex cryo-em dataset without replications. However, two half data analysis was performed to validate and generate the FSC curve. CryoSPARC algorithm uses the two half data analysis to evaluate if the data set is reliable and consistent between the two half data sets as convention. All attempts at this replication were successful. |
| Randomization | For cryo-em, images and particles were randomly picked by Relion, EMAN2, CryoSparc and et ac. And the final density maps were validated based on two randomized half datasets to avoid bias. |
| Blinding | No blinding was applied since no group allocation was perfromed. |

# Reporting for specific materials, systems and methods

We require information from authors about some types of materials, experimental systems and methods used in many studies. Here, indicate whether each material, system or method listed is relevant to your study. If you are not sure if a list item applies to your research, read the appropriate section before selecting a response.

## Materials & experimental systems

| n/a | Involved in the study |
| --- | --- |
| ☒ | ☐ Antibodies |
| ☒ | ☐ Eukaryotic cell lines |
| ☒ | ☐ Palaeontology and archaeology |
| ☒ | ☐ Animals and other organisms |
| ☒ | ☐ Clinical data |
| ☒ | ☐ Dual use research of concern |
| ☒ | ☐ Plants |

## Methods

| n/a | Involved in the study |
| --- | --- |
| ☒ | ☐ ChIP-seq |
| ☒ | ☐ Flow cytometry |
| ☒ | ☐ MRI-based neuroimaging |

## Plants

| | |
| --- | --- |
| Seed stocks | NA. |
| Novel plant genotypes | NA. |
| Authentication | NA. |
